# Evaluating the use of citizens’ juries in food policy: a case study of food regulation

**DOI:** 10.1186/1471-2458-13-596

**Published:** 2013-06-19

**Authors:** Julie Henderson, Elizabeth House, John Coveney, Samantha Meyer, Rachel Ankeny, Paul Ward, Michael Calnan

**Affiliations:** 1 School of Nursing and Midwifery, Flinders University, Adelaide, SA, Australia; 2 Discipline of Public Health, School of Medicine, Flinders University, Adelaide, SA, Australia; 3 School of History and Politics, University of Adelaide, Adelaide, SA, Australia; 4 School of Social Policy, Sociology and Social Research, University of Kent, Canterbury, Kent, UK

**Keywords:** Citizens’ juries, Deliberative democracy, Food regulation, Food sponsorship, Children sporting events

## Abstract

**Background:**

Deliberative engagement techniques and citizens’ juries are touted as means of incorporating the public into policy decision-making, managing community expectations and increasing commitment to public health policy. This paper reports a study to examine the feasibility of citizens’ juries as a means of collecting data to inform public health policy related to food regulation through evaluation of the conduct of a citizens’ jury.

**Methods:**

A citizens’ jury was conducted with a representative sample of 17 South Australians to explore their willingness to consider the proposition that food and drink advertising and/or sponsorship should be banned at children’s sporting events.

**Results:**

The results showed that, in relation to the central proposition and evaluation data from the jury, opinion on the proposition remained comparatively stable. Most jurors indicated that they thought that food and drink sponsorship and/or advertising at children’s sporting events would have little or no effect on altering children’s diet and eating habits, with the proportion increasing during the jury process. Jurors were given evaluation sheets about the content of the jury and the process of the citizens’ jury to complete at the end of the session. The evaluation of the citizens’ jury process revealed positive perceptions. The majority of jurors agreed that their knowledge of the issues of food and drink sponsorship in children’s sport had increased as a result of participation in the citizens’ jury. The majority also viewed the decision-making process as fair and felt that their views were listened to. One important response in the evaluation was that all jurors indicated that, if given the opportunity, they would participate in another citizens’ jury.

**Conclusions:**

The findings suggest that the citizens’ jury increased participant knowledge of the issue and facilitated reflective discussion of the proposition. Citizens’ juries are an effective means of gaining insight into public views of policy and the circumstances under which the public will consider food regulation; however a number of issues need to be considered to ensure the successful conduct of a citizens’ jury.

## Background

### Deliberative public engagement

This paper explores the value of deliberative pubic engagement as a means of gauging public opinion to inform food policy. Since the 1990s, authors [1-3] have identified a growing interest in methods which promote deliberative public engagement in policy making. Deliberative public engagement is a distinctive approach to involving people in discussions about policies and other value-laden issues [4], including ethical matters [5], which has its roots in deliberative democratic theory [6-9]. It is described as a “talk-centric democratic theory” in which the legitimacy of government is associated with policy and public accountability to citizens [2] (p. 317). This approach aims to give participants from diverse backgrounds and with a range of values and attitudes ample time and adequate information (without privileging the views of so-called ‘experts’) to enable them to consider and discuss an issue in depth before forming their views, and to carefully weigh reasons for and against a particular proposal. It emphasizes that outputs need not be simple answers about which policy would be preferred, for instance, but may well take the form of questions that remain open for participants and which would need to be resolved before they can endorse (or reject) a particular option. As such, it is a research method that can provide greater depth of knowledge than can be provided by alternative research methods such as survey research or focus groups, because the goal is to allow citizens to weigh evidence, discuss and debate potential policy options, and arrive at a mutually-agreed decision. Complete consensus may not be possible but the aim is to generate a decision that members of the collective can ‘live with’.

These methods have gained popularity given that the public is better educated, more sophisticated and less deferential to authority than in the past [10]. The methods are also claimed to be an antidote to political participation for individual purposes, and they endeavour to foster citizen participation as members of a broader collective, dovetailing with ideals of active citizenship [11,12]. In a review of literature on the use of deliberative techniques in health, Mitton et al. [3] note that they are most commonly reported in relation to public health issues. These methods are pertinent to public health because they have potential to empower participants through participation in and knowledge of health policy making processes [13]; involve community members in decision making about resource allocation [3] and priority setting [14]; manage community expectations [1]; and increase public commitment to policy decisions [15] and understanding community attitudes to perceived roles in self-management of health [16].

This paper explores the efficacy of a particular method of deliberative public engagement, the citizens’ jury, through a case study of a jury in Adelaide, South Australia, in December 2011. The main purpose of the jury was to gauge public views on the regulation of food and drink marketing and advertising at children’s sporting events. The jury also was used to trial methods and to determine the most effective means of conducting citizens’ juries in relation to public perceptions of food regulation, with reference to the key criteria for judging efficacy: representativeness, procedures and processes, quality of information, and outcomes. These 4 criteria have been recommended by Abelson et al. [4]. Representativeness is important to ensure that citizen participation is widespread and that issues of equity are addressed. Procedures and processes ensure that the jury’s deliberations are fair and responsive, and thus give rise to legitimacy. The quality of information used in the jury process is crucial, and needs to endure a breadth as well as depth of knowledge on the part of the witnesses. Finally, outcomes refers to the extent to which public input results in policy output; in other words, the extent to which the jury’s finding are incorporated into public policy considerations. Although there have been several studies of citizens’ juries with regard to public health policy, this mechanism has not yet been widely utilized in conjunction with food regulation. As this domain raises quite different issues particularly because of its intersection with people’s everyday lives as well as with issues associated with parenting and family decision-making, this study represents an important pilot in this domain.

### What is a citizens jury?

A citizens’ jury is a process in which a broadly representative group of citizens are brought together to discuss an issue that is of interest to the general community [17]. This model was developed in the United States (US) and Germany. In the United Kingdom the Institute for Public Policy Research (IPPR) has used it for a range of regional and national policy health issues [18]. The IPPR use 12–16 jurors while the Jefferson Center in the US recommends 18–24 members. Other citizens’ juries in the UK have also used 16 members [14,16]. Citizens’ juries are based on the premise that, if equipped with information and time, members of the public should be able to reach a decision on issues that they may have previously known little about [19]. Specialists with knowledge of the issue under discussion (witnesses) are selected to present information to citizens, generate discussion and answer any questions jurors may have [20]. The jury is given time to deliberate on the issue and it is assisted by facilitators who encourage debate and guide discussion [18]. The jury then forms recommendations on the issue that can be taken to policy makers for consideration in the policy-making process [19].

Citizens’ juries offer a unique opportunity for members of the population to engage in discussion and make recommendations for future policy making [14,19]. The advantages of the method include the involvement of members of the public, and the opportunity to gauge public reaction and opinion to policy during the policy-making process [21]. Specifically, jurors drawn from the general population are presented with evidence from specialists on an issue of policy, deliberate with fellow jury members and make their informed recommendations on the issue [22]. Potential considerations with the method include costs associated with venue hire, catering, staff wages and participant reimbursement [3,23], while a considerable amount of time is required to develop the jury, including identification and recruitment of jurors and specialist speakers. Despite these possible limitations, citizens’ juries are a unique public democracy mechanism that can yield rich data on public perceptions and recommendations on an issue of policy.

### Considerations in establishing a citizens’ jury

While deliberative democratic approaches are designed to encourage two-way interaction between policy makers and the public there are a number of factors that can inhibit this interaction. The first is related to sampling, recruitment and the representativeness of the jury. Gooberman-Hill et al. [23] argue that citizens’ juries generally should be composed of people from diverse backgrounds to ensure a range of opinions. Mitton et al. [3] identify three recruitment strategies: purposive sampling, self-selection and random sampling. Purposive sampling ensures that underrepresented voices are included in the jury, leading to greater equity, but this potentially ignores the impact of power relations based on symbolic power within the jury [23]. Recruitment through self-selection of participants is easier and more cost effective; however it can lead to homogeneity of participants and lack of diversity of views [12]. The relatively small size of juries means that random sampling may lead to panels that are inclusive but not representative [12]. This can be overcome through stratified random sampling and attention to the demographic characteristics of participants [24].

A second set of issues relates to the deliberation process. Procedural factors that need to be considered include structuring opportunities for participation; provision of adequate time for discussion [4]; pre- and post-testing to discern attitude change; effective facilitation [12]; and selection of witnesses who can represent opposing viewpoints [24]. Leadership style is particularly pertinent, with Ryfe [12] suggesting that the leader should establish group norms by outlining rules for equality, inclusiveness and civility at commencement and providing cues about how to act within the group throughout the process. In addition, Lenaghan et al. [14] argue that the definition of the question for the jury is crucial to its success.

A third consideration is the outcome of the deliberation process. Citizens’ juries can be understood as a consultative process that provides public input for policy makers [12]. Lenaghan et al. [14] argue that there are two models of citizens’ juries: a ‘deliberative model’ which involves broad ranging questions which may guide policy makers; and a ‘decision making model’ whereby the jury has a clear set of options from which to choose. The authors argue that both processes improve democratic deliberative processes, but the latter may improve the legitimacy of policy decisions. A number of authors [3,12,23] note there can be tension between lay perspectives and the realities of policy making. While citizens’ juries have the capacity to alert the public to these realities, they can also create an expectation that the consultation process will inform policy [12]. The reality of policy making is that public views are only one source of data among many. In making decisions, policy makers draw on research findings, experience, habits and tradition together with their own personal and political judgements [3]. As such, Mitton et al. [3] found that few studies formally evaluate the effectiveness of the deliberative public engagement process. However, Abelson et al. [4] suggest that evaluation can be addressed through the extension of data analysis beyond description of policy recommendations to a critical evaluation of the processes of deliberation. Lenaghan et al. [14] provided an evaluation of how their citizens’ jury operated, which was built into the development and operationalization of the citizens’ jury within this paper.

### Adelaide jury: background and topic

A research team from Flinders University, with associates from the University of Adelaide and the University of Kent who were experienced with citizens’ juries, developed and facilitated a citizens’ jury. The team was interested in pilot testing this model of citizen involvement using a current public health issue.

The chosen topic was the recent debate about food marketing to children and its influence on childhood overweight and obesity. In Australia it has been documented that 25% of children aged 5 to 14 years are overweight or obese. Contributing to these rates are environments which promote obesity (also known as ‘obesogenic’ environments) [25], one example of which is the sponsorship and advertising of unhealthy foods and beverages at children’s sport, through which children are exposed to the marketing of unhealthy food and drinks [26].

Investigations into the effects of sponsorship on children suggest that sponsorship has an impact on children’s recall and their product attitudes and food preferences [27]. This is problematic because the peak state organizations for children’s sport in South Australia rely on sponsorship from food and beverage companies and it is estimated that 92% of this promotion involves unhealthy products [26]. The significance of this issue has been identified by the World Health Organization which has recommendations on the marketing of foods and non-alcoholic beverages to children, including restricting the promotion of unhealthy food and beverages at sites where children gather, such as sporting activities [28]. Currently in Australia there are no regulations that restrict the promotion of food products to children through sponsorship [29].

In Australia there are calls for government intervention and policy to regulate food marketing to children, including pressure from key organizations and interest groups such as the Coalition on Food Advertising to Children and the Australian Council on Children and the Media. The views of the general public regarding policy development and reform, however, are under-represented. Therefore the specific question addressed by this citizens’ jury was “Should food and drink sponsorship of, and advertising at, children’s sporting events be banned?”. The project was given approval from the Social and Behavioural Ethics Committee at Flinders University, South Australia.

## Methods

### Recruitment

A broadly representative group of 20 participants was recruited by a market research company from a database of over 40,000 people. This is the same process as that used in other citizens’ juries [14,16]. We sought people without previous experience of citizens’ juries. The sample was stratified by socio-economic background, gender and political preference to ensure diversity, and there were people with and without children. Three participants withdrew prior to the sitting of the Citizens’ Jury, leaving 17 jury members (see Table 1).

**Table 1:** Participant demographic details

Name*	**Age**	**Gender**	**Occupation**	Socio-economic Status of suburb**	**Children Yes/No**	**Political preference**
Jill	28	Female	Student & emergency care worker	High	No	Labor
Matthew	28	Male	Plumber	High	No	Labor
Danielle	32	Female	Office manage	Low	No	Liberal
Nancy	31	Female	Factory worker	High	Yes	Labor
Darryl	37	Male	Council worker	High	Yes	Labor
Michelle	48	Female	Accounts manager	Middle	Yes	Liberal
Luke	45	Male	Fuel tanker driver	High	Yes	Liberal
John	60	Male	Retired	Middle	Yes	Liberal
Corinne	64	Female	Nurse	Low	No	Labor
Hayley	24	Female	Home duties	Low	Yes	Liberal
Noah	27	Male	Fitter & turner	Middle	No	Labor
Ben	35	Male	Teacher	Middle	No	Labor
Adam	36	Male	Landscaper	High	Yes	Labor
Dominic	42	Male	Occ health officer	Middle	Yes	Labor
Louise	56	Female	Home duties	High	Yes	Liberal
Jenny	55	Female	Real estate agent	N/A	Yes	Liberal
Henry	79	Male	Retired IT consultant	Low	No	Liberal

*Names have been changed to protect identities.

**Based on Socio-economic Index for Area (SEIFA).

Specialists in the areas of children’s health, sport and economics were identified from the networks of the research team. These specialists were invited to be witnesses and present information to the jury regarding the topic and their knowledge of it. Two witnesses were selected to present information in favour of the ban on food and beverage sponsorship and advertising at children’s sport and two to present information opposing the ban. Each witness was asked to provide five short points to the research team a week before the jury session to ensure their material addressed the issues effectively and genuinely represented divergent views.

### The process

A jury may meet over the course of 4 days [14,16], or 6–10 weeks [19]; Mitton et al’s review found that 40% of juries met on one occasion [3]. This jury met for one session lasting three hours. It was co-ordinated by two facilitators who had experience working with citizens’ juries. The jury began with a briefing on the format of the session and group norms. Jurors were polled with a series of questions and asked to select answers which best represented their opinion (see Table 2). These polls were conducted using interactive audience software and handsets, whereby graphs of the jurors’ answers were generated immediately following responses to enable pre- and post-testing of attitudes. The questions selected for the polls were chosen to address the circumstances in which a ban on sponsorship of and advertising at children’s sporting events would be supported. Specifically, whether location of the sporting events, and the type of food and drink sponsor mattered to jurors, or whether they believed in a blanket ban on sponsorship/advertising. Further questions were also used to gauge juror’s perception of the influence of sponsorship/advertising on children’s participation in sport and its effect on children’s diets. Lastly jurors were asked about how effective they believe such a ban would be in the fight against childhood obesity.

**Table 2:** Jury poll questions

**Poll questions**	**Possible answers**
1. Should food and drink sponsorship/advertising be banned at children’s sporting events?	∙ Yes, banned at all sporting events
	∙ Banned at selected events
	∙ No, not banned at any events
2. Food and drink sponsorship/advertising should be banned at:	∙ School and community sporting events
	∙ Private sporting events
	∙ All sporting events
	No sporting events
3. Which types of foods and drinks should be banned from sponsorship/advertising at children’s sporting events?	∙ All foods and drinks
	∙ All foods and drinks except fruit, vegetables and water
	∙ Foods and drinks high in salt, fat and/or sugar
	No foods/drinks should be banned
4. Do you see the effects of food and drink sponsorship/advertising at children’s sporting events as affecting the levels of participation in sport?	∙ Yes, very much
	∙ Quite a lot
	∙ Very little
	Not at all
5. Do you see the effects of food and drink sponsorship/advertising at children’s sporting events as altering children’s eating habits and diet?	∙ Yes, very much
	∙ Quite a lot
	∙ Very little
	Not at all
6. Do you think the Commonwealth Government should decide whether there should be a ban of food and drink sponsorship/advertising at children’s sporting events?	∙ Yes, it is the responsibility of the Commonwealth Government to regulate
	∙ No, it is parents’ responsibility to regulate children’s eating habits
	∙ No, industry should self-regulate
	∙ No, but the Commonwealth Government should provide information to parents and children about the risks associated with specific unhealthy foods and drinks
7. In the fight against obesity in children, do you think that a ban on food and drink sponsorship/advertising would be:	∙ Very effective
	∙ Reasonably effective
	∙ Barely effective
	∙ Ineffective

The witnesses were given 10 minutes each for their presentations, with five minutes of question time. The jurors were re-polled after all the witnesses had presented and the responses from both polls were presented on graphs that were visible to the jury. Following a break for refreshments, the jurors were split into two groups, predetermined to ensure each group contained jurors of both genders, with and without children, and with a range of ages, political preferences and socio-economic status. Each group had a facilitator to guide discussion and to identify the group opinion on the ban proposal. The rationale for splitting the group was to maximize the opportunity for each jury member to contribute to the deliberation, which was made easier by the smaller groups. The groups then reunited and a spokesperson from each group discussed their recommendations and the reasoning behind them. Further discussion as one group enabled an overall verdict to be reached, although there was some contention among a few of the jury members. The jurors were then re-polled for the final time and graphs of the previous polls appeared following responses from each question. Evaluation forms regarding the process and content of the jury were completed by jurors at the end of the session.

## Results

The results that follow present the finding from the jury in relation to the central proposition and evaluation data from the jury, used to gauge the efficacy of deliberative engagement methods in developing nuanced responses to public health policy.

### Electronic polling: tracking the impact of deliberation

Jurors were polled three times over the course of the jury: at the beginning of the session (Poll 1), following speaker presentations (Poll 2), and finally after the deliberation (Poll 3). Results from each poll were logged electronically, and the recording and relaying of this data to jury participants immediately after each question provided an opportunity to study the impact of deliberation and voting patterns on participants’ views by observing how voting changed over the course of the session.

The central proposition of the jury was “Should food and drink sponsorship of, and advertising at, children’s sporting events be banned?” Opinion on this proposition remained comparatively stable. Participants indicated that food and drink sponsorship and/or advertising should not be banned at children’s sporting events (~50% at each poll), or only banned at selected events (35% at each poll). The jurors were also questioned about what types of food and drink should be banned from sponsorship and/or advertising at children’s sporting events. Participants indicated greatest support for banning only foods and drinks high in salt, fat and or sugar (Poll 1: 41%, Poll 2: 45%, Poll 3: 50%). This was followed by the opinion that no food or drinks should be banned (Poll 1: 29%, Poll 2: 41%, Poll 3: 31%), while there was least support for the banning of all foods and drinks except fruit, vegetables and water (Poll 1: 29%, Poll 2: 12%, Poll 3: 19%). None of the participants at any stage of polling voted that all food and drinks should be banned from advertising or sponsorship at children’s sporting events. There was little variation across polls in response to a question on the impact of food and drink bans on childhood obesity. Approximately 75% of jurors at each of the three polls thought that a ban would be ineffective or barely effective in the fight against childhood obesity. The remaining jurors thought it would be reasonably effective, but only one juror in the final poll voted that the ban would be effective in the fight against obesity in children.

Changes in participant views were more evident in relation to the topics specifically covered in presentations. Jurors were asked about how effective they thought a ban on sponsorship and/or advertising would be on altering children’s eating habits. Most jurors at each poll indicated that they thought that food and drink sponsorship and/or advertising at children’s sporting events would have little or no effect on altering children’s diet and eating habits, with the proportion increasing during the jury process (Poll 1: 59%, Poll 2: 77%, Poll 3: 88%). The proportion of jurors who believed that a ban would impact children’s eating habits ‘quite a lot’ or ‘very much’ fluctuated from poll to poll (Poll 1: 41%, Poll 2: 5%, Poll 3: 12%). There were also marked differences in participants’ views about whether the loss of food and drink sponsorship and/or advertising at children’s sporting events would affect participation in those events, with changes most evident at the deliberation stage (Poll 1: 18%, Poll 2: 23%, Poll 3: 65%).

### Jury recommendations and verdict

In addition to polling, jury deliberations within the groups and discussion of the final verdict were recorded and transcribed to capture the conditions under which participants would support the proposition. Jury members were, for the most part, against the proposal that all food and drink sponsorship of, and advertising at, children’s sporting events should be banned, although there were a few jurors who were strongly in favour of the ban. There were several reasons given by the jury for their positions on the ban. Jurors explained that participation in sport comes with associated costs including uniforms and membership fees. They suggested that sponsorship and advertising often subsidise this cost, and if a ban were introduced parents would have to absorb the extra cost, which may not be possible for some families. Jurors agreed on the importance of the funding provided by sponsorship and advertising to sporting events and clubs, so instead of banning them altogether they suggested having some form of regulation over how sponsorship could be provided. For example, if McDonald’s wanted to provide encouragement awards or vouchers they should be for a healthy choice e.g. salad and water rather than an unhealthy one. Alternatively, jurors suggested that non-food or beverage company sponsors could be sought, including real estate agents and other local companies.

Jurors also questioned the effectiveness of the ban on improving children’s eating habits. They noted that food and drink companies use aggressive television advertising targeted at children and therefore, children will still exposed to these products regularly via television even if they are banned from sporting events. Consequently jurors believed that the ban would not make much difference in tackling overweight and obesity in children.

In contrast, those jurors in support of the ban reasoned that there should not be a link between sporting heroes and brands because children are influenced by this. Instead, they thought that funding should come from other sources. These jurors stated that the government cannot control what parents buy but they can have some control over advertising. They cited the example of government regulation of tobacco advertising and reduced rates of smoking.

### Evaluating the jury process

Following Abelson et al. [1], jurors were given evaluation sheets about the content of the jury and the process of the citizens’ jury to complete at the end of the session. The evaluation revealed positive perceptions of the process. The majority of jurors (82%) agreed that their knowledge of the issues of food and drink sponsorship in children’s sport had increased as a result of participation in the citizens’ jury. The majority (95%) also viewed the decision-making process as fair and felt that their views were listened to (94%). Some feedback was provided about the jury processes. This feedback included allocating extra time for jury deliberations, providing a broader range of speakers and providing more facilitation in the larger group deliberation. One important response in the evaluation was that all jurors indicated that, if given the opportunity, they would participate in another citizens’ jury.

## Discussion

Citizens’ juries engage members of the public in issues of interest to the general community [21]. They also provide the opportunity for jurors to increase their knowledge on a given issue and make informed decisions and policy recommendations [14,16,19]. This study yielded information on the opinions of members of the public about the banning of food and drink sponsorship of children’s sports. Data were collected through electronic polls and through analysis of the jury’s deliberations and verdict. In addition, jury members were asked to provide feedback on the process. This paper contends that deliberative engagement techniques, and in particular citizens’ juries, provide a means of collecting nuanced data about the circumstances under which a representative sample of the public will accept increased regulation of food and drink sponsorship of, and advertising at, children’s sporting events. As such, they provide a means of engaging the public and incorporating their views in debate about food regulation and more broadly about public health policy particularly in contested domains where everyday values are at issue (e.g., perceptions of good parenting).

This jury was held in part to test the efficacy of citizens’ jury methods for examining questions relating to food regulation and hence it was important to evaluate the processes that accompanied the jury’s deliberations. It is not easy to determine success of citizens’ juries [3]. Abelson et al. [4] state that evaluation of citizens’ juries usually consists of a brief discussion of lessons learned from deliberation; however they argue that four aspects of the jury process should be evaluated, namely the representativeness of the sample, procedures and jury processes, the quality of information, and outcomes. The reminder of this paper will explore each of these issues in turn with recommendations made for future conduct of citizens’ juries.

### Representativeness of the sample

Underpinning deliberative public engagement is the assumption that the legitimacy of policy making can be enhanced through citizen participation in decision making [12]. This process is associated with identifying a wide range of options but also with equity [4]. As such, obtaining a sample that is representative of the wider community is an important consideration in recruitment for citizens’ juries. Following Abelson et al. [4], Elwood & Longley [16] and Lenaghan et al. [14], in this study we used a stratified sample to attract participants with a variety of geographic, demographic and political characteristics. The sample was obtained through a market research company. Table 1 demonstrates that participants came from a range of socio-economic backgrounds (occupational status and suburb); ages and voting preferences. Both genders were represented, as were people with and without children. However, the majority of participants were currently employed or had retired from the workforce and there was limited ethnic diversity. This probably reflected the population pool drawn on by the market research company. Gooberman-Hill et al. [23] warn against the exclusion of under-represented voices. As a consequence, it may be necessary to purposefully sample for under-represented groups.

### Procedures and jury processes

Abelson et al. [4] recommend examination of procedural aspects of the jury with respect to legitimacy, responsiveness and fairness. As indicators of successful juries they highlight opportunities for decision making, time for discussion, opportunities to challenge and question expert views, and mutual respect, which can be expressed as an equal chance to contribute to proceedings [24]. The rules of engagement should also be outlined at the commencement of the jury [12]. This citizens’ jury consisted of one 3-hour session. Mitton et al. [3] argue that the holding of one rather than several sessions may prevent the development of trust that arises from long-term engagement. They note, however, that in approximately 40% of the studies they reviewed the juries met on only one occasion. Given the nature of the proposition in our study, it is doubtful whether multiple sessions would have provided further insight. This view is supported by Abelson et al. [1], who identify the nature and scale of the question as an aspect of context that should be considered in establishing citizens’ juries. Despite this, it is evident that further time was needed for deliberation. Opportunities were scheduled for questioning of experts and deliberation, and while changes of opinion during the process and the nuanced views presented at verdict show that both processes had an impact on jury views we recommend further time be allotted for the deliberation process. The expert opinions presented provided a stimulus for initial deliberations. However, it is during the deliberation process that critical discussions intensify and jurors progress towards a final verdict. Extra time during the deliberation process would allow jurors to engage in more critical discussion which is important for moving towards consensus. However, we caution against allowing deliberation to continue beyond the point of consensus as we did identify that at times conversation was dominated by select members of the group and deliberations became cyclical. This finding also points to the need for an experienced facilitator who can recognise when the deliberation process should conclude.

### The quality of information

Decisions on how information is presented and interpreted are essential in protecting the impartiality of the process [4,14]. Blamey et al. [24] argue that this can be ensured through the selection of witnesses who represent divergent viewpoints while Ryfe [12] highlights the importance of impartial leadership to facilitate discussion. One issue raised in respect to witness testimony in this study was the extent to which the testimony directly addressed the proposition. In short, it was found that testimony that addressed the questions included in the electronic poll had greater impact. Another unexpected finding from jury feedback was that the sex and profile of each expert affected the delivery of, and response to, his or her presentation. In our study both witnesses supporting the proposition were female and both witnesses opposing it were male. In selecting witnesses the aim is to minimize the extraneous differences in experts’ presentations that may influence opinion to allow the jury to focus on the argument presented, so consideration should be given to the gender of witnesses.

### Outcomes

A final consideration is the outcome of the jury. Abelson et al. [4] argue for evaluating outcomes in terms of the extent of incorporation of public input into decision making, participant satisfaction, knowledge development and achievement of a degree of consensus. Feedback on this aspect of process was generally favourable, with the majority of the jurors indicating that they gained knowledge of the issue and that that they would participate in other citizens’ juries if given the opportunity. Furthermore, a degree of consensus was reached about the conditions under which a ban would be acceptable to this group. Some authors [3,12] question the extent to which citizens’ juries can inform policy development in any context and because this was a pilot study there were limited opportunities for incorporation of jurors’ views into policy. Abelson et al. [1] identify the culture of the sponsoring organization as an important contextual factor in the success of citizens’ juries. This project was supported by university funding, limiting links with government and policy makers. Ideally, working in conjunction with government provides opportunities for greater knowledge exchange.

## Conclusion

This paper has outlined the principles of deliberative engagement and of citizens’ juries, drawing upon a case study about food regulation to demonstrate that citizens’ juries provide a valuable means of gauging public opinion to inform public health particularly with regard to food regulation and policy. In addition, they have the capacity to provide information as to the circumstances under which the public will accept new policy directives. Four aspects of practice were addressed in relation to successful citizens’ juries: the representativeness of the sample; impartiality of the procedures; quality of information presented; and outcomes and use of data. The paper has drawn upon the experiences of the authors in conducting a citizens’ jury to add to this literature. We conclude that the use of citizens’ juries can provide rich data to policy makers with findings demonstrating that a ban on food and drink sponsorship and/or advertising at children’s sport may not be supported by the general public and that other regulations may be more acceptable to the public.

## Competing interests

The authors declared that they have no competing interests.

## Authors’ contributions

JC conceived of the study, and participated in its design and coordination and helped to draft the manuscript. All authors were members of the team that executed the research. EH was project manager and wrote first draft of manuscript. JH completed draft for manuscript submission. All authors read and approved the final manuscript.

## Pre-publication history

The pre-publication history for this paper can be accessed here:

http://www.biomedcentral.com/1471-2458/13/596/prepub
